# Early Echocardiographic Predictors for Atrial Fibrillation Propensity: The Left Atrium Oracle

**DOI:** 10.31083/j.rcm2306205

**Published:** 2022-05-31

**Authors:** Lavinia-Lucia Matei, Roxana-Mihaela Popescu, Andreea Catarina Popescu, Șerban Mihai Bălănescu

**Affiliations:** ^1^Cardiothoracic Medicine Department, University of Medicine and Pharmacy Carol Davila, 020021 Bucharest, Romania; ^2^Department of Cardiology, Elias Emergency University Hospital, 011461 Bucharest, Romania

**Keywords:** left atrium dimensions left atrium function, left atrium remodeling, left atrium reverse remodeling, atrial fibrillation

## Abstract

Atrial fibrillation (AF) results from structural and electrical remodeling of the atria, primarily of the left atrium (LA); therefore, LA changes, both anatomical and functional are recognized as proarrhythmic markers with a powerful prognostic value. Being widely available and noninvasive, echocardiography is used to monitor LA form and function in clinical practice. Early functional (electrical) remodeling of the LA precedes anatomical alterations. Impaired LA functions and reduced atrial compliance due to atrial fibrosis may be evaluated using novel echocardiographic techniques, such as tissue Doppler analysis and speckle tracking. Functional evaluation of the LA conveys prognostic information about the risk of AF, as the severity of the impairment is an independent predictor of new-onset AF and AF recurrence. However, specific parameters are still investigated for incorporation into algorithms to predict future AF occurrence. The aim of the review is to summarize echocardiographic parameters, their predicting value and applicability in practice.

## 1. Introduction

Atrial fibrillation (AF) occurs in up to 4% of the general population and is considered a global pandemic with a rising prevalence. AF is a major health problem since the estimated lifetime risk of an individual to develop AF is about 25% [1, 2, 3, 4, 5].

Functional remodeling determines LA dysfunction, highlighted by decreased contractility when using volumetric assessment or strain analysis. LA enlargement, with consequent decrease in its mechanical function, represents a maladaptive structural and functional “remodeling” that promotes electrical remodeling [6, 7].

Cardiac imaging plays a pivotal role in the evaluation and management of patients with AF. Imaging enables the identification of associated conditions that lead to AF development and perpetuation while providing information on AF’s effect on the LA. The challenge for clinical cardiologists is to detect LA functional remodeling at an incipient stage, prior to anatomical changes.

Transthoracic echocardiography (TTE) is the first line imaging modality and is of paramount importance when assessing the LA in patients with AF, as it is a noninvasive, reproducible, and widely available technique. TTE gives information about LA dimensions and size changes, and also about the LA hemodynamics. It provides information about cardiac anatomy and global function, chamber dimensions, intracardiac pressure gradients and valvular status. It unmasks atrial fibrosis effects that determine LA anatomical remodeling and impairment of LA functions. Different variables obtained by TTE were evaluated to predict AF in multiple studies, with an emphasis on LA dimensions and more recently LA function. The purpose of this review is to summarize these findings and their applicability in clinical practice.

## 2. Role of LA in AF — Pathophysiology

The pathophysiology of AF is complex and not fully understood. The pathogenesis of this tachyarrhythmia involves a complex relationship between triggers, substrate, and modulators, that mainly refer to ionic and structural remodeling, neuro-humoral contributors, and genetic predisposition [4, 8]. The electrical trigger arises from the left atrium (LA) and is afterward disseminated to both atria. For the arrhythmia to be sustained, a modified atrial substrate is mandatory. The hypothesis that “AF begets AF” is demonstrated by studies showing that structural remodeling is the main mechanism that contributes to generating and perpetuating this tachyarrhythmia [2, 6].

In order to characterize this complex association of anomalies, the term “atrial cardiomyopathy” was introduced [8]. Atrial cardiomyopathy is defined as a cumulus of structural, architectural, contractile, and electrophysiological changes in the atria [8]. The remodeling process is progressive and time-dependent, as a response to different variables that include electrical, mechanical, and metabolic factors [4].

Structural remodeling occurs due to increased interstitial fibrosis, while atrial enlargement develops consequently. Fibrosis is mediated at a cellular level by various factors as a response to pathological conditions, cardiovascular risk factors, and aging [7]. Age dependency of AF is emphasized by arrhythmia prevalence of 10% in the general population of patients older than 80 years [9]. Not only individual factors, but also multifactorial processes due to diverse interactions between cellular, neurohormonal and inflammatory mediators, in association with genetics and individual predisposition are implicated in AF substrate generation [9].

Atrial fibrosis is of major importance in the development of AF, due to conduction abnormalities with proarrhythmic risk [10, 11]. Fibrotic myocardial tissue is defined by disarranged myocytes and increased collagen with expanded extracellular interstitial space. Not only the degree, but also the characteristics of LA fibrosis may determine a good prediction of AF development and recurrences [10, 11]. Delayed enhancement magnetic resonance imaging for assessing myocardial fibrosis is a non-invasive technique, with good intra and interobserver reproducibility also for LA area and volumes [12]. Several studies show that the degree of fibrosis strongly correlates to recurrent arrhythmias and constitutes a predictor of sinus rhythm maintenance [13, 14, 15]. Atrial fibrosis is a cause of AF, but also a consequence of AF. Invasive techniques refer to the electrophysiological approach to identify areas with low voltage and abnormal electrograms. Electro-anatomical mapping suggests that fibrosis precedes AF development [16, 17]. Historically, AF triggers were thought to appear around the pulmonary veins’ ostia [16, 17]. Nonetheless, changes in atrial substrate might generate novel induction sites. Low voltage zones are predictors of AF recurrences after AF ablation [16] and additional low voltage zones- based substrate modification after pulmonary vein isolation will improve the outcome [17].

A better understanding of AF mechanisms and role of cardiac fibrosis might help the development of personalized therapeutic approaches. Correct measuring of the degree and types of LA fibrosis might improve clinicians’ decision-making for AF patients.

## 3. Left Atrial Size in AF

Increased LA dimensions are associated with adverse cardiac outcomes and are directly correlated with the incidence of AF [18]. Dimensions of the LA are important markers of structural remodeling that may also indirectly provide information about the arrhythmogenic substrate. LA enlargement develops as a consequence of atrial fibrosis, thus its dimensions were proposed as the first anatomical change prior to AF emergence. Two-dimensional transesophageal echocardiography (TOE). LA diameter, area and volume help quantify LA anatomical remodeling, and are used as predictors of AF onset and recurrence [19]. The LA is also a marker of diastolic dysfunction and increased LA pressures, both associated with the development of AF [20, 21]. Different measurements of LA dimensions were studied in patients with different clinical patterns of AF [18, 19, 20].

**Fig. 1.**
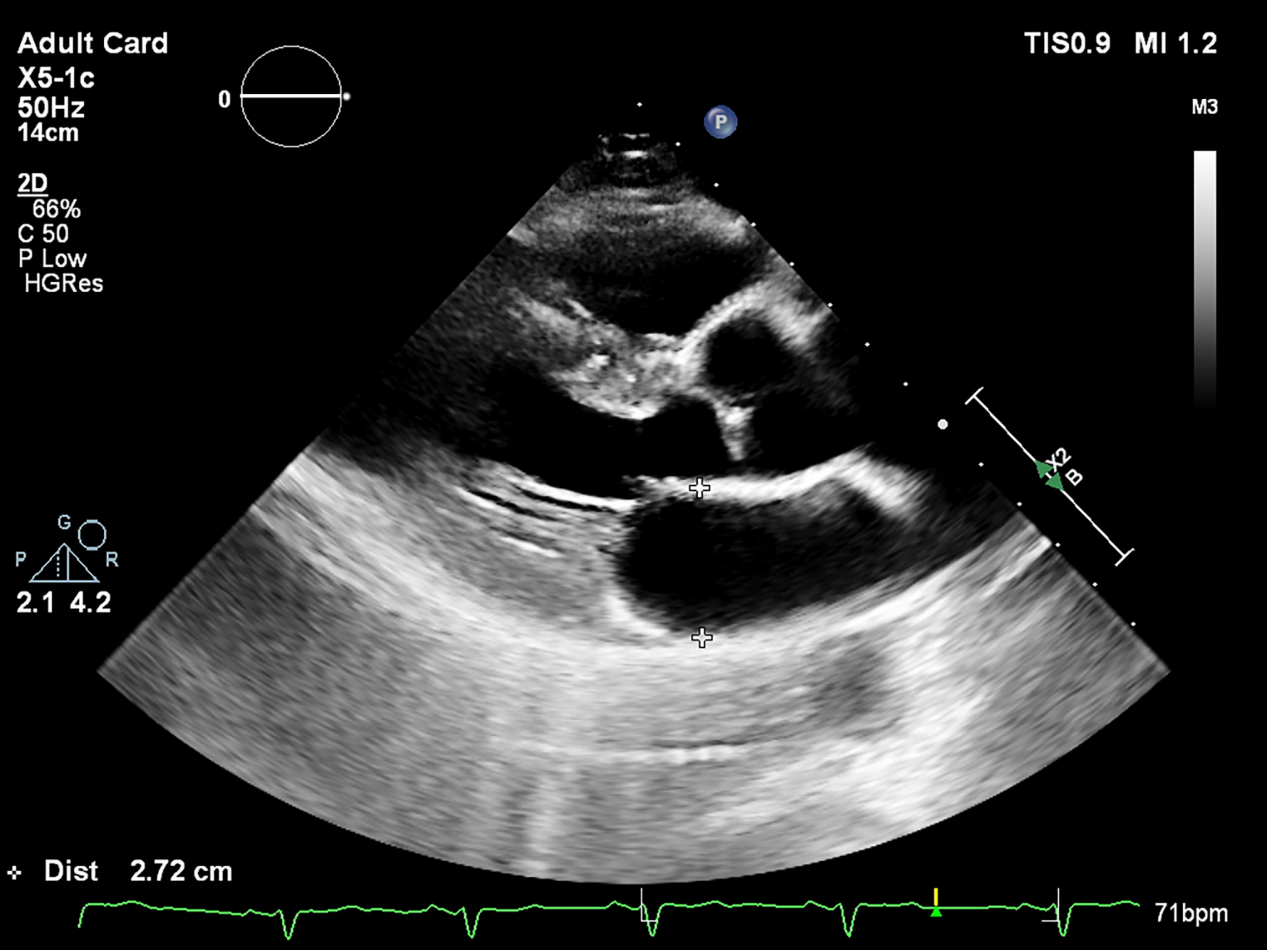
** Measurement of the LA diameter (end-systole, inner edge to inner edge) in the parasternal long-axis view by bidimensional echocardiography**.

**The LA antero-posterior (AP) diameter** is the most evaluated parameter in patients with AF. The recommended measurement technique uses the long-axis view in bidimensional echocardiography (2D-E), perpendicular to the LA posterior wall, from inner edge to inner edge (Fig. 1). The latest chamber quantification recommendations suggest that the M-mode evaluation should no longer be used. The LA AP diameter was intensely evaluated in relation to AF development. An increased diameter, with a cut-off value of 40 mm, was associated with a higher risk of AF [22, 23]. This diameter should not be used solely, as it doesn’t represent the true LA size [24]. Moreover, the latest statements define a normal LA AP dimension less than 40 mm in males and 38 mm in females, with an indexed value of less than 23 mm in both genders [24].

The AFFIRM study (RAte Control versus Electrical Cardioversion for Persistent Atrial Fibrillation) shows that LA diameter is correlated with the risk of AF recurrence after spontaneous, chemical or electrical conversion to SR [25]. The LA size correlates with the presence and frequency of paroxysmal AF episodes [26]. An AP LA diameter over 50 mm was a prognostic predictor of recurrences after a first AF ablation, irrespective of the pattern of AF (paroxysmal or non-paroxysmal) [27]. Tops *et al*. [28] also determined that a LA AP diameter of less than 45 mm, associated with reverse remodeling after radiofrequency catheter ablation, is prognostic of a good outcome in the long term.

Longitudinal (superior-inferior) and transverse (mediolateral) diameters of the LA are measured in a dedicated 4-chamber view. Predominant enlargement of the LA in one of these diameters will alter the geometry and the volumes concordantly, even if the AP diameter remains in normal ranges.

**Fig. 2.**
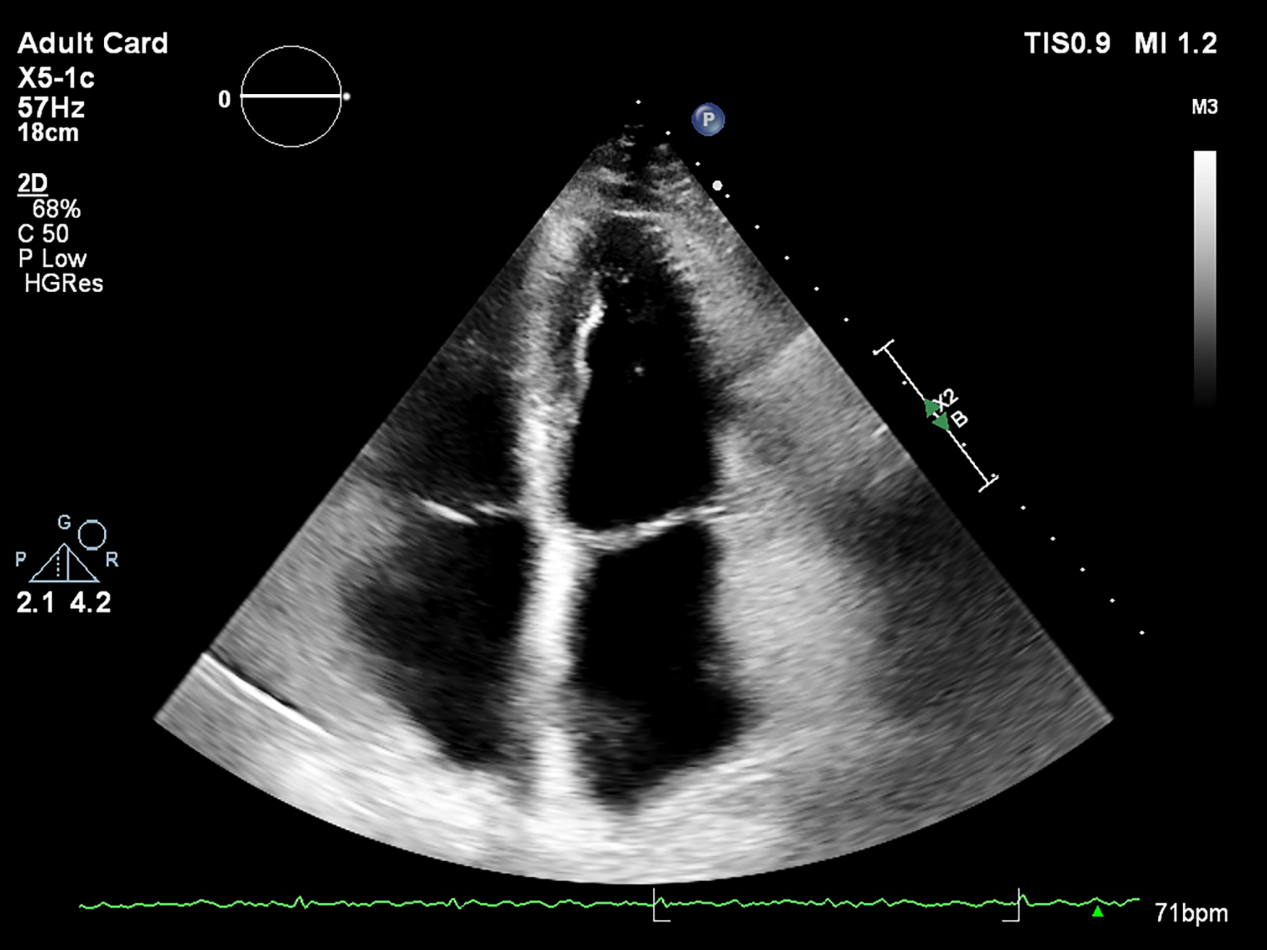
**Bidimensional echocardiography – 4 chambers view, maximum left atrium dedicated view**.

**Measurement of the LA volume** is preferred over linear or area assessments, because LA remodeling is asymmetrical, and therefore volumetric measurement is more precise in assessing LA enlargement [29, 30, 31]. The volume should be measured using the area-length method or modified biplane disk summation, in dedicated views, avoiding foreshortening of the LA long axis (Fig. 2). Pulmonary veins and LA appendage should be excluded from the measurements [24]. TTE allows measurements of all LA volumes: (1) maximal LA volume is measured just before the opening of the mitral valve in left ventricular end-systole, (2) minimal LA volume is measured at the closure of the mitral valve in end-diastole, (3) LA passive volume consists of pre-atrial contraction volume that is measured at the onset of the P-wave on the ECG. The method depends on correct positioning and angulation of imaging planes, requiring dedicated views and implying geometric assumptions about LA spatial geometry.

**The left atrium area** can be evaluated using planimetry in the apical 4- and 2-chamber view. The normal reported value for the LA area is under 20 ${\mathrm{cm}}^{2}$. Volumes are easily calculated and due to robust literature studies on prognosis, volumes exceed the importance of area reporting [32]. Using only LA diameters and area measurements, more than 50% of patients may be misclassified regarding the presence and degree of LA dilation [29].

An enlarged LA indexed volume is defined as more than 34 mL/$m^{2}$, irrespective of gender [24]. A 2D-E LA indexed volume of 34 mL/$m^{2}$ has a sensitivity of 70% and specificity of 91% to predict AF recurrences [33]. Several meta-analyses demonstrate that increased LA volumes are independently predictive of AF development [34, 35] recurrences after AF ablations [36], or progression to persistent AF forms [37].

**Fig. 3.**
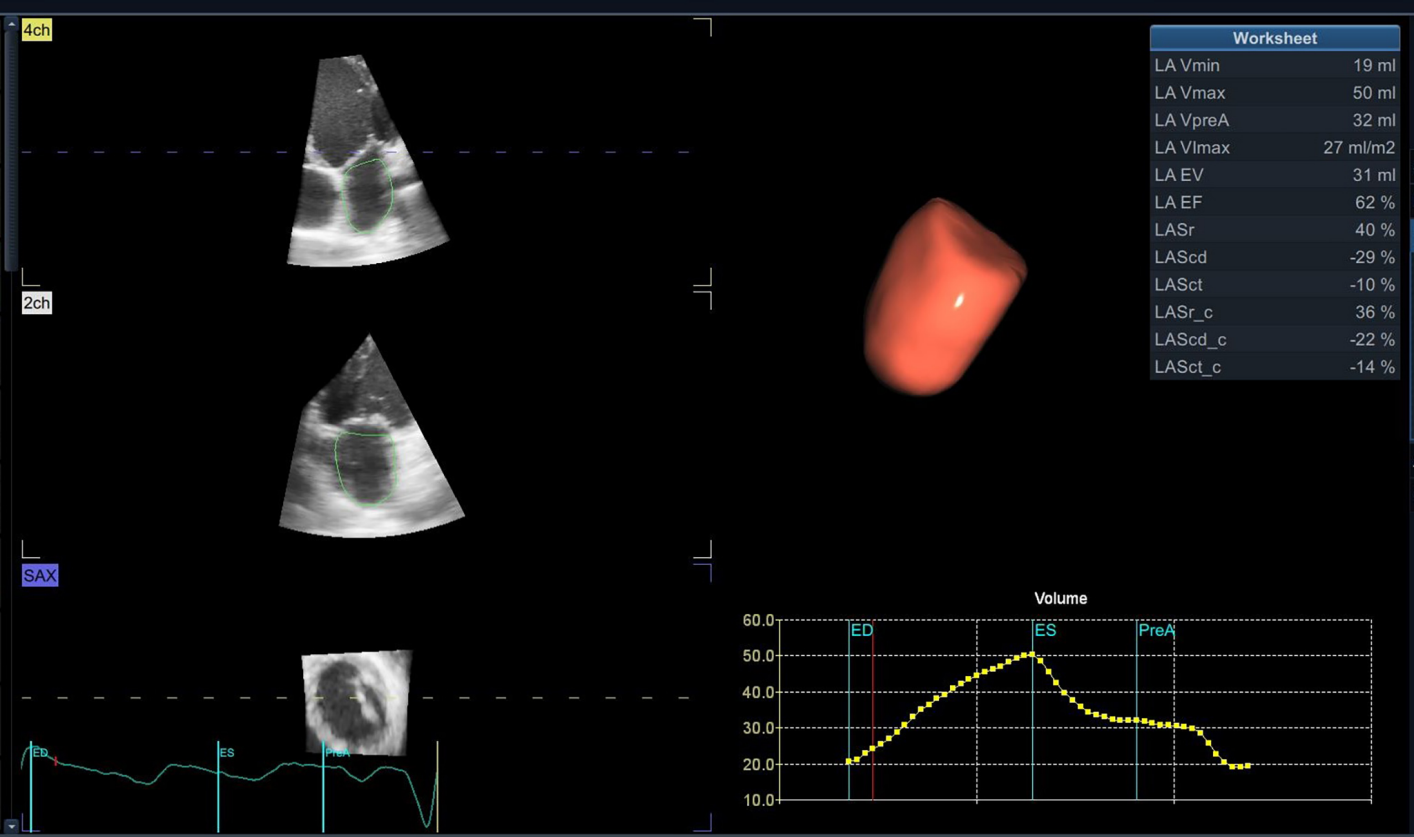
**Left atrium tridimensional volume and function using 3D-E speckle tracking with a dedicated software**.

LA enlargement usually occurs as a result of persistent chronic pressure overload. The LA volume evaluation represents an integral part of evaluating ventricular diastolic function. Bi-dimensional imaging obtained by 2D-E does not reflect the true LA size, and three-dimensional echocardiography (3D-E) should be superior for accurate measurement. Atrial volumes by 3D-E (Fig. 3) correlate with 2D-E assessment, but values are higher, as 2D-E underestimates LA volumes in comparison with other imagistic techniques (especially cardiac magnetic resonance, which is the gold standard). Most studies evaluated LA volumetry using dedicated left ventricular programs. Though data is still lacking for normal ranges, a study in healthy volunteers by Badano *et al*. [38] determined a maximum LA index volume in 3D-E of 43 mL/$m^{2}$, and a minimum LA volume of 18 mL/$m^{2}$ compared to a volume of 14 mL/$m^{2}$ in 2D-E.

The maximum volume is the most evaluated parameter associated with AF [30, 31, 39], but more recent studies concentrated on LA minimum volume as a marker of prognosis [40, 41, 42, 43]. Appleton *et al*. [44] showed that minimum LA volume is more closely related to left ventricular filling pressures evaluated invasively, compared to maximum LA volume. In patients with paroxysmal AF, LA remodeling evaluated by both 2D-E and 3D-E is strongly associated with AF episodes, that are best predicted by LA minimum volume [40]. Minimum LA volume seems to be an independent predictor of a first tachyarrhythmia episode [41, 45]. Nevertheless, the LA maximum volume is more reproducible in terms of inter-observability when measured in 2D-E [41], and even 3D-E [38].

When therapeutic strategies are applied in the early stages of the development of paroxysmal episodes [46, 47], by lowering the AF burden and maintaining the sinus rhythm (SR), the LA will present a reverse remodeling process [25, 28, 48]. Atrial fibrillation ablation, especially in patients with “lone AF”, in the absence of structural cardiac disease, will determine reverse atrial remodeling [49]. Reports define LA reverse remodeling as a decrease of more than 15% in LA index volume, compared to baseline values, assed by TTE [28]. The process of reverse remodeling is reported with 2D-E studies [25, 28, 46, 48], but also with 3D-E studies. Even if 3D-E is not largely available in clinical practice, it seems to be a feasible and reproducible imaging method also for in-training echocardiographers [50], while providing more accurate values of the true LA dimensions, as compared with cardiac magnetic resonance evaluations [51, 52]. A limitation for 3D-E refers to patient cooperation, high imaging quality, and frame-stitching over consecutive regular cardiac cycles, making evaluation for AF patients during arrhythmias inconclusive [45].

In conclusion, the most useful anatomical parameter to characterize AF propensity should be LA volume and mainly LA indexed volume. Assessment of LA volume is best performed by 3D-E, should both ultrasound hardware and software be available and echocardiographic views accurate.

## 4. Left Atrial Function in AF

Functional changes of the LA are more subtle and may occur prior to the gross anatomical changes described above. They are more difficult to identify in clinical practice but should be searched for in order to properly study AF propensity in a given patient. The LA has three different functions during the cardiac cycle: (1) it acts as a ‘booster pump’ when it contracts in late left ventricle diastole, (2) then as a ‘reservoir’ during systole, (3) and as a ‘conduit’ during early ventricular diastole.

**LA passive volumes** consist of pre-atrial contraction volume, measured at the onset of the P-wave; minimal LA volume, measured at the closure of the mitral valve in end-diastole; and maximal LA volume, measured just before the opening of the mitral valve in end-systole. (1) LA active volumes are LA reservoir volume or LA filling volume, (2) LA conduit volume or LA passive emptying volume, and (3) LA contractile volume. The functions may be calculated using volumetric assessments, or novel echocardiographic techniques. Routine indications for measurements are not yet implemented [53], even if alteration of LA phasic functions are described and studied in many cardiovascular conditions, with an emphasis on AF [54]. An important limitation of LA functions using volumetric differences is that these parameters are derivative and incorrect evaluation of 2D-E LA volumes could potentially determine a modification of emptying fractions [55].

Evaluating LA volumes implies a time-consuming method with low reproducibility if we consider manual delineations of the LA myocardium. Newer software may facilitate LA volumes acquisition due to automated myocardial tracking techniques. Okamatsu *et al*. [56] demonstrated a close correlation between 2D-E manually traced LA volumes measurements and speckle-tracking derived volumes of the same images. Myocardial tracking techniques were more reproducible and twice as fast compared with manual evaluation.

Functional measures of the LA are good predictors of AF in the general population. The Copenhagen City Heart Study concluded, after evaluating almost two thousand individuals for over 11 years of follow-up, that besides the LA volumes (maximum and minimum), the** LA ejection fraction** (calculated as the difference between LA maximum volume and LA minimum volume, divided by LA maximum volume) was also an independent but rather weak predictor of AF occurrence, with an HR = 1.03 (95% CI = 1.02–1.04) for every percentual decrease [45]. Both passive and active LA functions seem to be age-related (r = 0.8, *p *< 0.001) [56], just like LA volumes, as concluded by “The Multiethnic Study of Atherosclerosis” (MESA) after evaluating a healthy multiethnic community-based population aged 53–94 years [57]. Nonetheless, even if maximum and minimal LA volumes increase over time, with significant acceleration due to aging and incidental AF, LA ejection fraction remains unchanged over a period of 10.4 years [58].

**Left atrial function index** (LAFI) emerged as a surrogate for an easier evaluation of the LA function, as it is considered as a composite echocardiographic parameter for LA structure and function, adjusted to left ventricular function. Independent of the cardiac rhythm, LAFI characterizes LA function by combining LA volume and emptying function with left ventricle stroke volume (LAFI = LA ejection fraction × LVOT – VTI (left ventricle outflow tract velocity time integral) / maximum LA volume indexed to body surface) [59]. Studies show that LAFI is associated with the development of recurrent AF, heart failure, and stroke. In the Framingham offspring study, by Sardana *et al*. [60] LAFI proved to detect subtle pathological changes in LA structure and function, clinically relevant for predicting AF in patients with cardiovascular disease, or strokes in patients with coronary artery disease [61].

In conclusion, evaluating the LA function using manually volumetric assessment is not routinely recommended in daily clinical practice, as these parameters have a high interobserver variability. LA indexed volume may, on the other hand, be useful in predicting outcome, as they are influenced by the degree of LA fibrosis.

### 4.1 Left Atrial Evaluation Using Pulsed Wave Doppler Echocardiography

Besides quantification of LA changes, TTE provides information about LA hemodynamic function. Pulsed wave (PW) Doppler at the mitral leaflets and at the pulmonary veins can give a hint on LA function, by noninvasive estimation of the intra-atrial pressures.

**Fig. 4.**
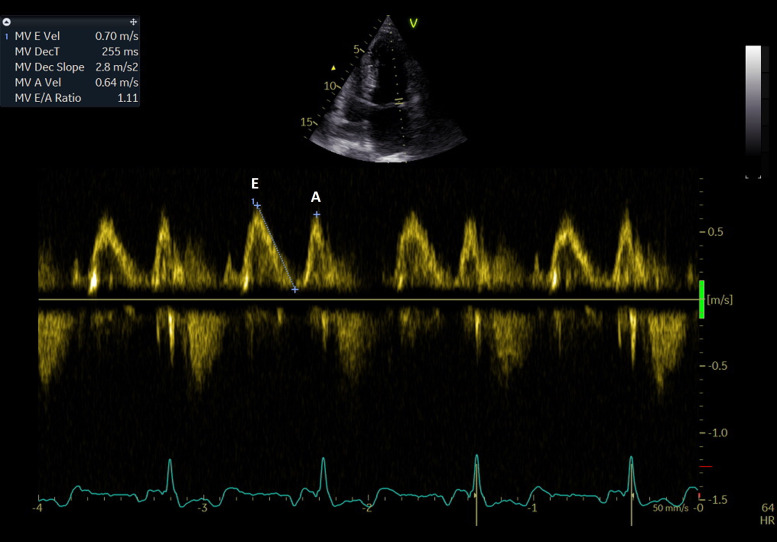
**PW Doppler evaluation of the transmitral diastolic flow. E wave – early diastolic filling of the LA; A wave – late diastolic filling determined by atrial contraction**.

**The mitral inflow pattern** is evaluated using PW Doppler, with the sample volume placed at the tips of the mitral leaflets in the apical four-chamber view (Fig. 4). The evaluation demonstrates the passive LA filling during ventricular diastole (the E wave) and the active late filling due to atrial contraction (the A wave). The E/A ratio in healthy, euvolemic young adults is typically higher than 1. The ratio depends on age and the E wave decreases in older individuals, resulting in a ratio below one.

In patients with AF, beat-to-beat variability and absence of the A wave make the E/A ratio impossible to assess. Moreover, the hallmark of LA dysfunction is the loss of atrial contraction in AF: the A wave disappears, but restoration of SR results in its reappearance. The loss of atrial contraction, which accounts for up to 30% of left ventricle filling, will determine a reduced ventricular volume, while creating a volume overload for the LA. This results in progressive LA dilation, determining myocyte disarray and fibrosis. The A wave velocity is evaluated as a surrogate of LA contraction function, as full recovery of mechanical activity does not occur immediately after successful SR restoration. Thus, A wave velocity after cardioversion to SR from AF is low, as far as LA systolic function does not promptly recover. This phenomenon is described as “LA stunning”. Left atrium stunning may persist between 24 hours for the paroxysmal pattern [62] and up to 3 weeks [63] after cardioversion, depending on the duration of AF [64].

A higher than 2 E/A ratio in patients with SR indicates a restrictive pattern of LV diastolic function. This is associated with an E wave deceleration time lower than 140–160 msec and an IVRT lower than 50 msec. This pattern is indicative of increased LV end-diastolic pressure, and it predicts high LA pressure and volume. A restrictive diastolic mitral flow pattern is correlated with mortality in patients with heart failure [65]. A high E/A ratio that results from restrictive LV conditions should not be confused with that observed after AF cardioversion when A wave velocity is low due to atrial stunning.

**Fig. 5.**
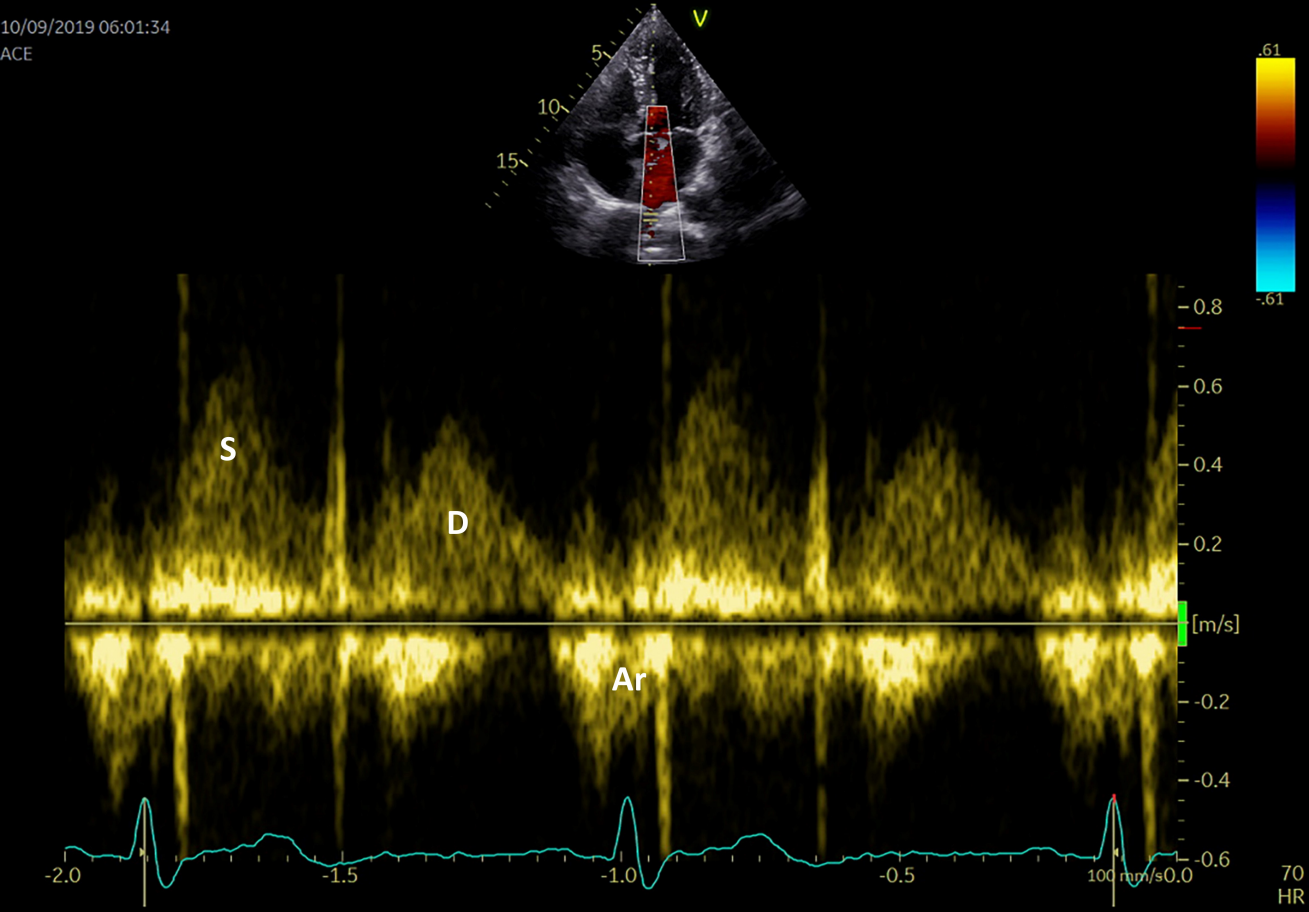
**Pulmonary venous flow using PW Doppler echocardiography S systolic flow, D diastolic flow, Ar atrial reversal**.

**The pulmonary venous flow** can also be evaluated using the sample pulse volume into one of the pulmonary veins in the apical 4-chamber view (Fig. 5). The peak systolic velocity consists in 2 waves: S1—the LA active relaxation and S2—the LA filling or reservoir function, while the peak anterograde diastolic velocity ‘D wave’ relates to the LA conduit function, and the peak retrograde diastolic velocity ‘Ar wave’ to the LA booster pump function. Although many physiological variables may affect the pulmonary venous flow aspect and velocities (age, preload, heart rate, left ventricular function), there is a significant correlation between LA pressure and S2 peak velocity, while the S2/D ratio can evaluate the LA reservoir function [66].

A reduction in systolic pulmonary peak velocity is determined by increased LA pressures and is associated with increased frequency of paroxysmal AF and propensity for AF recurrence following restoration of SR. It is also correlated with a reduced LA appendage velocity flow and a higher risk of thrombus formation [67].

Pulmonary veins evaluation is not usually part of a standard echocardiographic examination, therefore prognostic information should be obtained using the mitral inflow pattern. This parameter also discloses data about the function of the left ventricle. While it can characterize LA function using hemodynamic LA capacity, the PW Doppler evaluation of the LA does not offer robust predictors of outcome.

### 4.2 Left Atrium Appendage Velocity

**Fig. 6.**
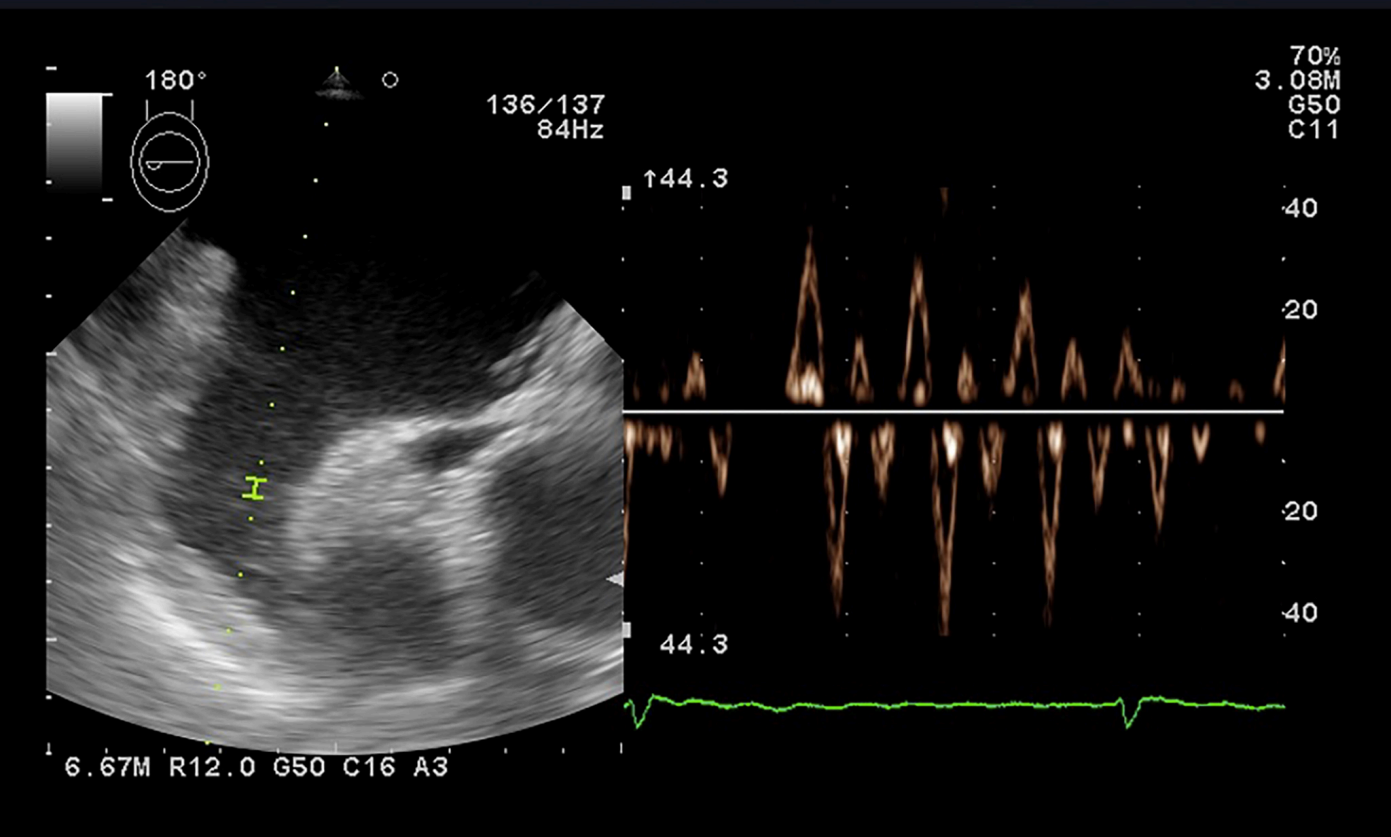
**PW Doppler evaluation of the LAA flow showing low velocities in a patient with atrial fibrillation**.

In clinical practice, preprocedural observation of LA appendage (LAA) by echocardiography is mainly performed to detect thrombus formation. Transesophageal echocardiography may guide management in patients with AF, as LAA velocity (Fig. 6) may be evaluated as an independent predictor of paroxysmal AF development and should be integrated as a novel predictive parameter. A LAA velocity of less than 40 cm/s correlates with high thromboembolic risk and lower chances of successful cardioversion and SR persistence. Increased LAA velocities (>40 cm/s) are associated with a higher likelihood of SR persistence after cardioversion [68, 69]. Even during an AF episode, the velocity may guide further treatment options, as an increased velocity could identify candidates for successful electric or pharmacological cardioversion [68, 70]. Atrial fibrillation recurrence after ablation is more common in patients with lower LAA velocity, but the thresholds are yet to be established [71, 72, 73]. He *et al*. [73] evaluated patients with paroxysmal AF who underwent ablation and determined a higher risk of AF recurrences for a velocity under 39.2 cm/s (sensitivity 75%, specificity 82%). In persistent AF patients, patients with recurrences showed lower LAA velocities (23.3 ± 7.2 cm/sec versus 33.3 ± 15.1 cm/sec), as Kanda *et al*. [72] showed in their study, with a cut-off value of 28 cm/s (sensitivity 62%, specificity 69%). Following cardiac surgery, the odds of postoperative AF decreased by 11% for each 10-unit cm/s increase of appendage velocity [74].

### 4.3 LA Function Evaluation with Tissue Imaging Echocardiography

**Fig. 7.**
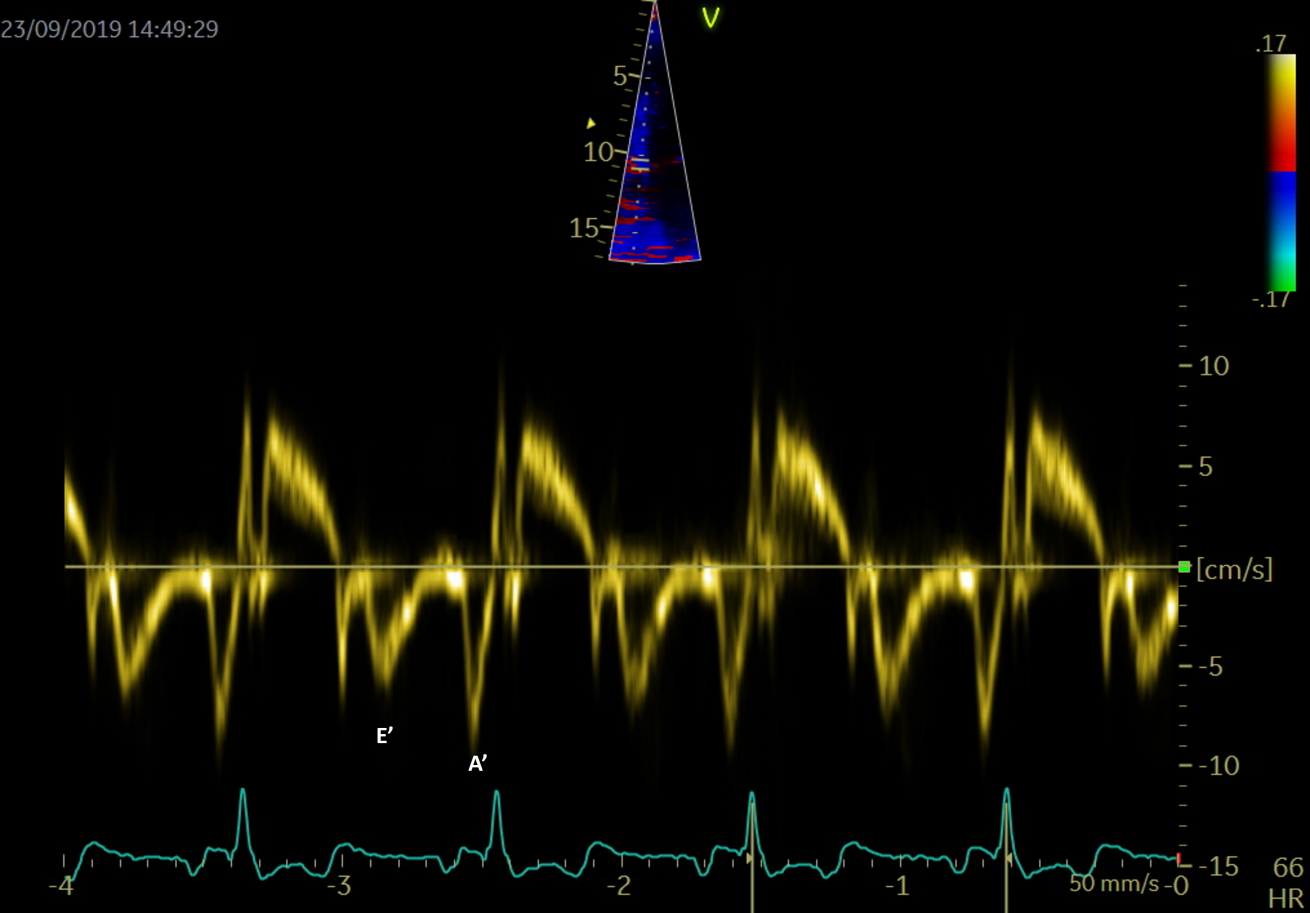
**PW Tissue Doppler evaluation at the level of the septal mitral annulus for evaluation of E’ and A’**.

PW tissue Doppler imaging (TDI) is performed in the apical 4-chamber view, with the sample volume placed at the level of the mitral annulus, to evaluate the left ventricular wall motion (Fig. 7). The E/E’ ratio, correlates to LA pressures and is associated with an increased risk of late AF recurrence after catheter ablation. The risk is 3.32 times higher for a threshold over 13.25 [75].

TDI can also assess an atrial segment of interest, and measurements are usually done at the lateral wall level. The peak velocity in late diastole correlates to atrial contraction (A’) and is a rapid and accurate marker of atrial function. Hesse *et al*. [76] demonstrated a good correlation between A’ and LA fractional area and volume change in quantifying LA systolic function.

In AF patients there is a decreased compliance of LA walls, as during AF the reservoir and conduit function are impaired, and the booster pump function is lost. The S wave corresponds to the reservoir function, the lateral atrial E’ wave to the blood conduit, and the atrial A’ wave to the atrial contraction function.

The LA activity closely relies on the left ventricle functional parameters, and changes in left ventricle function and left atrial mechanics are independent and interrelated. Diastolic dysfunction and AF have many common risk factors, including aging [77]. It is known that most diastolic parameters vary with age: E and E’ wave velocities decrease, while the A wave, the E wave deceleration time and E/E’ ratio increase depending on age [78].

Not only the presence, but also the severity of left ventricle diastolic dysfunction is independently predictive of newly developed AF [79]. Early diastole filling evaluated by tissue Doppler mitral annulus motion velocity (the E’ wave) is reduced in patients with diastolic impairment. In this context, the E/E’ ratio evaluates filling pressures of the LA and left ventricle stiffness. In patients with diastolic dysfunction, the E/E’ ratio is used as an independent predictor of AF [80]. The relationship between transmitral E velocity (related to LA pressure and left ventricle relaxation) and tissue Doppler mitral annulus velocity (E’ – reflects left ventricle relaxation) reflects atrial pressures, irrespective of left ventricular function. This ratio seems to have a clinical relevance in risk stratification in patients with AF. A ratio over 11.2 was determined as a predictor for early AF recurrences [55], while a higher ratio, over 13.25, was established as an independent predictor for late AF recurrences [75]. A septal E/E’ ratio over 15 was evaluated as an independent predictor of mortality in patients with AF [81]. Both lateral atrial E’ and the ratio E’/A’ show a good correlation with various diastolic dysfunction parameters and LA strain [82]. While E/E’ ratio has been validated as an independent predictor of evolution and recurrences in the evaluation of outcome in patients with AF, data regarding thresholds are still missing [37, 55].

Atrial electromechanical delay or LA dyssynchrony time can also be measured by TDI. It is a feasible method that can evaluate the presence and extent of LA remodeling in addition to conventional echocardiographic parameters. TDI PA peak time (PA peak - TDI) is defined as the time measured from the start of the P wave in lead II to the peak of A wave on the tissue Doppler tracing from the lateral LA wall. Left atrial asynchrony was demonstrated to be an independent predictor for AF recurrence after radiofrequency ablation [83].

Tissue imaging is mostly used in clinical practice to determine the E/E’ ratio, as this parameter gives information about the relation between the LA and the left ventricle.

### 4.4 Speckle Tracking Echocardiography of the Left Atrium

Speckle tracking echocardiography is an advanced imaging technique that estimates myocardial deformation, assessing LA functions. The region of interest is defined by the endomyocardial border and the epicardial border (the inner and the outer contour of the LA). The displacement of the speckled pattern is considered to follow the myocardial movement. The strain is defined as the percentage of change from the original dimension, while the strain rate measures the velocity with which myocardial deformation occurs, expressed as $S^{-1}$. The LA strain is studied only in the longitudinal plane, as the radial strain cannot be evaluated due to the thin LA wall. The strain analysis can be easily implemented to identify abnormalities in LA function that highlight LA fibrosis. Recent studies state that LA strain and strain rate correlate to the degree of LA fibrosis on late gadolinium enhancement MRI, while changes in LA functions may precede anatomical changes in patients with subclinical alterations of the substrate [51, 84, 85, 86]. Detecting early functional remodeling of the LA remains a challenge for the clinician. LA strain and strain rate are inversely related to LA fibrosis diagnosed by MRI and directly related to AF burden [87]. Patients with persistent AF have lower longitudinal strain in the mid-septal and mid-lateral walls, compared to patients with paroxysmal forms [87].

**Fig. 8.**
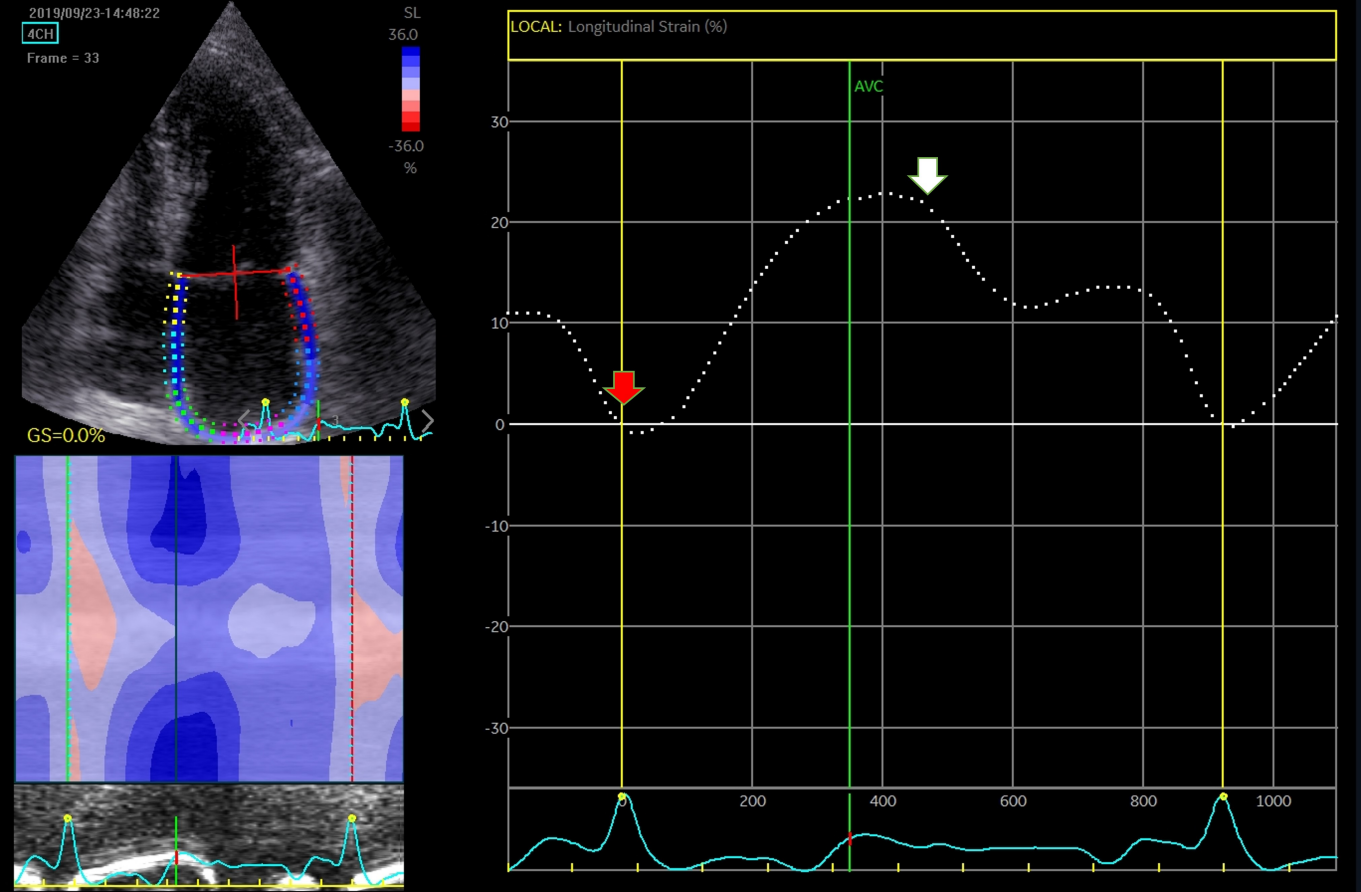
**Assessment of LA function by speckle tracking echo using the R-R interval – peak systolic LA strain (arrow)**. Four-chamber view depicting the region of interest (ROI, in the left). The curves represent the mean global LA longitudinal strains. The reference point was set at the onset of the R-wave. The total global strain is positive at the opening of the mitral valve (red arrow). Global strain at atrial contraction is also positive (white arrow). The total global strain is a sum of the negative global strain at atrial contraction (red arrow – at mitral valve closure) and the positive global strain (white arrow – at mitral valve opening).

**Fig. 9.**
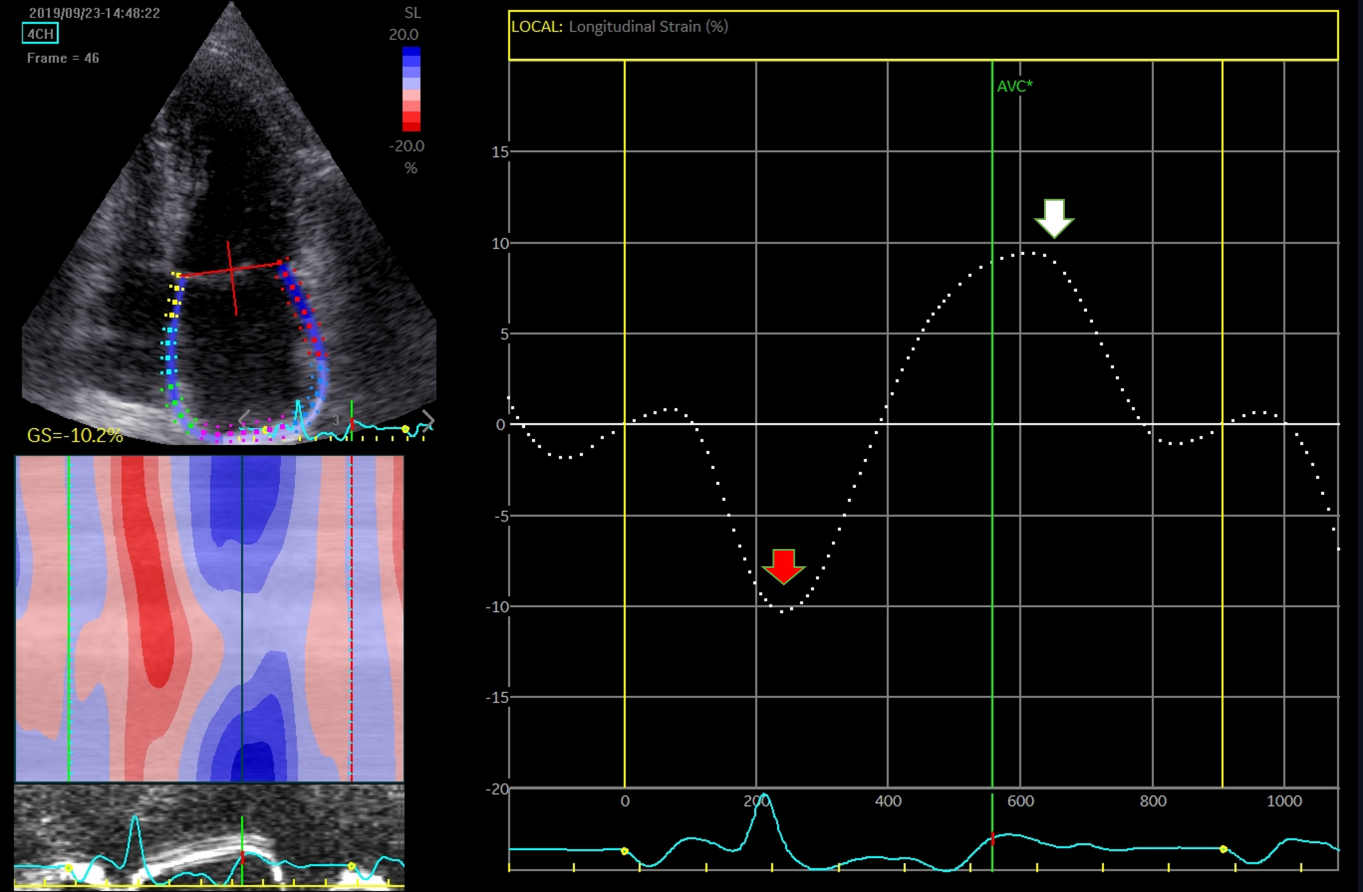
**Strain evaluation of the left atrium using the P-P interval**. Four-chamber view depicting the region of interest (ROI, in the left). The curves represent the mean global LA longitudinal strains. The reference point was set at the onset of the P-wave. The total global strain is a sum of the negative global strain at atrial contraction (red arrow – at mitral valve closure) and the positive global strain (white arrow – at mitral valve opening).

Strain analysis has the advantage of being a semi-automated, angle-independent technique. The need to manually track the LA walls and reposition the region of interest on each segment, makes this investigation time consuming and decreases reproducibility. Until recently, studies relied on a single software, and consensus and interchangeability between different software are still needed. Irrespective of these limitations, atrial strain is validated and correlates with the degree of fibrosis [88]. The assessment can address a 4-chamber view (6 segments) only, or both 4- and 2-chamber views (12 segments), in dedicated optimized views to record a maximized cross-sectional image of the chamber. The recommendations highlight exclusion of pulmonary veins and appendage orifice [53]. Interpretation of LA strain as global strain is advised, while using a single apical 4-chamber view to assess LA longitudinal strain is acceptable. Notably, the interatrial septum is more difficult to visualize. This is the reason why most studies disregarded the interatrial septal deformation and focused on lateral wall movement [89]. The LA septal strain is influenced by its fibromuscular composition and by right atrial pressure. In 2- chamber view, the problem of strain analysis depends on the LA appendage, which often compromises the recognition of speckles and deformation analysis [90]. The measurements may be interpreted using the QRS-complex [53] or the P-wave intervals [91], with similar reproducibility, but better feasibility and shorter time-to-analysis for the former (Figs. 8,9). Moreover, the QRS-complex interval has an advantage when assessing patients with arrhythmias, as AF [92].

The limitations of the method include the location of the LA, reduced signal to noise ratio, a thin wall, and the presence of pulmonary veins and LA appendage. These particularities make the LA strain analysis more difficult and time-consuming compared with left ventricle strain evaluation [90].

LA strain of the reservoir phase (LAS-r) corresponds to LA early diastole (peak atrial longitudinal strain - PALS); LA strain in the conduit phase (LAS-cd) corresponds to LA mid-diastolic emptying with its passive shortenings while LA strain in the contraction phase (LAS-ct) or peak atrial contraction strain (PACS) corresponds to LA systole with active myocardial shortening that produces the atrial contribution to ventricular filling. In normal individuals, the reservoir, passive conduit, and pumping phase account for 40%, 35%, and 25% of left ventricular filling, respectively [90]. There is no single measurement that can be used to determine LA function, and several parameters have been used in clinical studies. Nevertheless, the global function is best reflected by the reservoir strain [53, 54].

Strain measurements, LA reservoir, and conduit strain vary with age. They are significantly lower by the sixth decade, and between genders. During this time, the LA contractile strain improves, as a compensatory mechanism, and this mechanism is predominant in males [93].

Compared to SR patients, in patients with AF, the reservoir and conduit strain are decreased, while the atrial contraction is absent. The alteration of the reservoir function may be detected even before AF development, as a consequence of LA fibrosis and reduced compliance. Reservoir function by LA speckle tracking has an inverse linear relationship to cardiac magnetic resonance late gadolinium enhancement detecting fibrosis, thus evaluation of the function before a procedural approach may be a strong predictor of outcome [47]. After SR restoration, reverse atrial remodeling is reflected by an increase in LA reservoir function and passive conduit strain [94]. This improvement in the LA reservoir function may be approached as a marker of a successful outcome and could show its value in the early recognition of patients with AF recurrences [95].

A reduced LA active pump function independently predicts new-onset AF [96], while the LA strain is strongly associated with AF recurrences after ablation [97, 98] and may even predict evolution toward non-paroxysmal episodes [37]. Progression of paroxysmal AF to sustained episodes is predicted by different echocardiographic parameters as Yoon *et al*. [37] demonstrated on a cohort of approximately 300 paroxysmal AF patients, that not only refer to LA size (LA diameter >39 mm, LA volume >34.2 mL/$m^{2}$), but also measures of LA function (E velocity, E/A, and E/E’ ratio, LA expansion index and LA longitudinal strain and strain rate). In a group of patients with paroxysmal AF who underwent ablation, global strain and systolic strain rate in all segments and even average values were significantly reduced compared to control group patients [85], while the degree of reduction was an independent predictor of AF recurrences [99].

Restoration and maintenance of SR after AF cardioversion determine an improvement in LA contractile function, while LA reservoir strain improves mostly in patients that show LA reverse remodeling [97, 99, 100]. Nonetheless, catheter ablation, by applying radiofrequency lines that isolate the pulmonary veins, disorganize the substrate and therefore could alter the LA contraction strain. A lower LA strain and strain rate is dependent on the degree of myocardial scar. The extent of the scar formation after AF ablation is associated with a higher recurrence risk [101].

The speckle tracking derived **LA stiffness index** is a non-invasive surrogate of atrial fibrosis. It is calculated as a ratio between mitral E/E’ and LAS-r. Patients with AF have increased LA stiffness in comparison with control subjects, with higher values in patients with non-paroxysmal forms of AF [102]. Atrial fibrillation recurrences also tend to be more common in patients with an increased LA stiffness index [86, 103].

Certainly, LA structural and functional remodeling are closely correlated and depend on the extent of pre-existing fibrosis. Even if data shows that revers-remodeling of LA diameters and volumes occurs after SR restoration, it seems that LA ejection fraction and active booster pump parameters do not improve, and even show a decrease in patients with AF recurrence [104], while a lower LA ejection fraction may increase the risk of AF development [39].

The LA global strain can be evaluated using a single apical 4-chamber view and the QRS interval, while LA functions may provide additional information about prognosis and evolution in patients with AF.

Echocardiographic evaluation of LA structural and functional remodeling is a feasible non-invasive method, helpful in predicting outcome in AF patients.

## 5. Clinical Applicability

Classic 2D-E TTE has a central role in evaluating patients with AF, especially prior to restoration to SR. Although, LA anatomical remodeling may be preceded by structural changes, most of the time, evaluation is limited to LA dimensions. In this case, we support the importance of LA indexed volumes, with an emphasis on LA minimum volume. Tridimensional TTE adds prognostic information based on more accurate measurements, but the investigation is limited due to less availability and difficulty of correct acquisitions in patients with arrhythmias.

Speckle tracking offers valid measurements, that are less dependent on cardiac preload. Assessment of atrial strain could be challenging, due to location of the LA further away from the ultrasound, and the thin LA wall encumbers accurate tracing. Correct strain acquisitions and measurements need significant expertise and a longer duration of acquisitions. A reduction in atrial deformation during the reservoir phase should be considered an early marker of LA fibrosis, and in consequence a predictor of ablation outcome.

The end-game of early evaluation of the LA, in patients with AF or at risk for AF, is the LA reverse remodeling process that can be achieved through timely intervention.

## 6. Future Perspectives

We consider that TTE, both 2D-E and 3D-E should be implemented as basic evaluations in patients with high risk of AF onset, and in all patients after SR restoration. Echocardiography is shown to be a good non-invasive surrogate for the degree of LA dysfunction, with lower costs to cardiac MRI or electrophysiological studies. Periodical assessment may show LA functional and structural evolution (remodeling or revers-remodeling due to SR maintenance). As most parameters are evaluated at rest and given the fact that most patients exhibit symptoms during exercise, evaluation of the dynamic LA functions may be of future interest as a cornerstone in their therapeutic approach. This additional information may help tailor the personalized strategy of each patient, related to primary or secondary prevention of AF.

## 7. Conclusions

Left atrium evaluation by multiple echocardiographic methods, from basic 2D-E to 3D-E and speckle tracking provides diagnostic and prognostic data. There is evidence to support use of comprehensive assessment of both left atrium size and function in clinical practice to detect the risk of new-onset AF or AF recurrence.

TTE is largely available, compared to cardiac MRI or electro-anatomical invasive methods. A good comprehension of LA structural and functional parameters and their relation to LA fibrosis could be of use in general cardiology practice, when assessing a patient with AF.

The most simple and useful parameters for AF prediction address static measurements as the indexed LA volume (both maximum and minimum) and the E/E’ ratio. We consider that these measurements should be a part of the basic evaluation, while speckle tracking assessment would require a more prolonged examination by an experienced echocardiographer. Strain and strain rate measurements are a feasible, non-invasive and cost-efficient surrogate method of assessing LA fibrosis. Measurements of LA size corroborated with LA functions, using more complex parameters derived from strain imaging or tridimensional echocardiography could bring additional information in guiding physicians to manage AF patients even in the presence of a non-dilated LA. In this case, a personalized approach to future strategy should allow differentiating patients with a higher benefit from a precocious ablation, or an early reintervention after AF recurrences, in terms of SR maintenance and cardiac remodeling.

## Abbreviations

AF, atrial fibrillation; LA, left atrium; SR, sinus rhythm; TTE, transthoracic echocardiography; 2D-E, bidimensional echocardiography; 3D-E, three-dimensional echocardiography.
